# A simple method for gene phasing using mate pair sequencing

**DOI:** 10.1186/1471-2350-15-19

**Published:** 2014-02-06

**Authors:** Kendall W Cradic, Stephen J Murphy, Travis M Drucker, Robert A Sikkink, Norman L Eberhardt, Claudia Neuhauser, George Vasmatzis, Stefan KG Grebe

**Affiliations:** 1 Department of Laboratory Medicine and Pathology, Mayo Clinic, Rochester, MN 55905, USA; 2 Department of Molecular Medicine, Mayo Clinic, Rochester, MN 55905, USA; 3 Information Technology, Mayo Clinic, Rochester, MN 55905, USA; 4 Adavanced Genomics Technology Center, Mayo Clinic, Rochester, MN 55905, USA; 5 Department of Medicine, Division of Endocrinology, Mayo Clinic, Rochester, MN 55905, USA; 6 Department of Biochemistry and Molecular Biology, Mayo Clinic, Rochester, MN 55905, USA; 7 Biomedical Informatics and Computational Biology, University of Minnesota Rochester, 111 South Broadway, Suite 300, Rochester, MN 55904, USA

**Keywords:** Gene phasing, Compound heterozygosity, Haplotype, Next generation sequencing

## Abstract

**Background:**

Recessive genes cause disease when both copies are affected by mutant loci. Resolving the *cis/trans* relationship of variations has been an important problem both for researchers, and increasingly, clinicians. Of particular concern are patients who have two heterozygous disease-causing mutations and could be diagnosed as affected (one mutation on each allele) or as phenotypically normal (both mutations on the same allele). Several methods are currently used to phase genes, however due to cost, complexity and/or low sensitivity they are not suitable for clinical purposes.

**Methods:**

Long-range amplification was used to select and enrich the target gene (*CYP21A2*) followed by modified mate-pair sequencing. Fragments that mapped coincidently to two heterozygous sites were identified and used for statistical analysis.

**Results:**

Probabilities for *cis/trans* relationships between heterozygous positions were calculated along with 99% confidence intervals over the entire length of our 10 kb amplicons. The quality of phasing was closely related to the depth of coverage and the number of erroneous reads. Most of the error was found to have been introduced by recombination in the PCR reaction.

**Conclusions:**

We have developed a simple method utilizing massively parallel sequencing that is capable of resolving two alleles containing multiple heterozygous positions. This method stands out among other phasing tools because it provides quantitative results allowing confident haplotype calls.

## Background

The use of diagnostic gene sequencing has dramatically increased during the last two decades. However, accurate interpretation of sequencing data remains a challenge, despite technical advances. One common problem is uncertainty about the *cis*/*trans* status, or phase, of heterozygous variations. Properly phased genomic information is frequently required for accurate diagnosis of recessive genetic diseases. The scale of this problem is considerable, as indicated by a recent query of the Online Mendelian Inheritance in Man (OMIM) database which revealed over 250 recessive genes known to be associated with more than 1,100 disorders [1]. Unfortunately, Sanger sequencing, the most widely used technique and current gold standard, is incapable of separating phases without allele-specific capture or allele-specific amplification.

While this problem has long been recognized, a simple and effective solution has remained elusive. Computational methods have been developed to estimate haplotype sequences based on the individual’s genotype compared to a population [2], but they lack the resolution and accuracy needed for clinical use.

A more definitive approach for genetic phasing is based on manipulation of single chromosomes, either through cell hybrid systems, using conversion technology [3,4], or by means of size-exclusion devices [5]. While this strategy is perhaps the most reliable for generating accurate haplotype sequences, it is by far the most labor intensive approach. It is also error and failure prone, due to its lengthy, complex and technically difficult workflows.

More recently, the phasing problem has been tackled using massively scaled Next Generation Sequencing (NGS). Briefly, these methods depend on the creation of at least 100 libraries from each patient using techniques such as bacterial fosmid construction or multiple displacement amplification [6,7]. Libraries are indexed, pooled, sequenced and then computationally combined into two haplotype consensus sequences. While these methods are powerful for generating phased sequences for entire genomes, they are cumbersome, slow and currently expensive.

Since each of these approaches is in some way unsuitable for routine clinical use, current protocols for solving *cis*/*trans* questions typically involve testing of family members. This is a costly and time consuming undertaking that may still fail, if there is insufficient genetic diversity in the tested familial cohort. As an alternative, allele-specific PCR can be employed. However, the cost and effort required to design and validate assays makes this prohibitive in genes where there are many possible combinations of mutant positions.

Revisiting NGS techniques, with a view to creating a simpler solution than multiple indexed library sequencing, could provide an attractive solution to the phasing problem, in particular as NGS is now starting to replace Sanger sequencing in clinical applications. Because NGS methods are based on deriving sequences from a single molecule, one should be able to adapt the methodology for accurate phasing of genomic sequences. Most of the current platforms use a paired end (PE) protocol in which a string of sequence is read from either end of a larger DNA fragment. Since the reads come from opposite ends of the same fragment and are linked through a continuous strand of DNA, we refer to them as linked reads. Given their linked nature, any variations detected in the same fragment are *cis* to one another.

**Figure 1:**
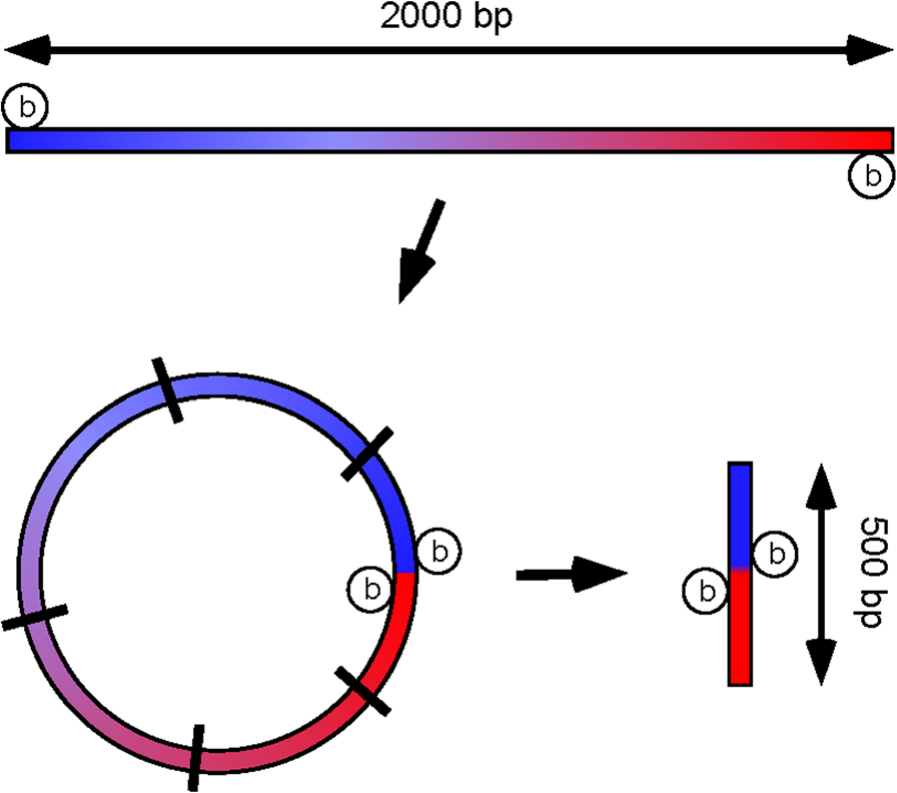
**Mate pair library preparation.** The MP protocol allows sequence information to be linked across greater distances than PE reads. Fragments of sheared DNA from a pool (500 – 5000 bp with an average of 2000 bp) are end-repaired using biotinylated nucleotides. Fragments are then self-ligated and all remaining linear DNA is removed by exonuclease treatment. Circularized DNA is fragmented again (black bars) to an average size of 500 bp and segments containing biotinylated junction points are isolated on streptavidin beads. In addition to fragments containing junction points, a portion of non-biotinylated DNA is co-purified and appears in the MP library as a subpopulation of PE reads. All fragments are end-repaired and indexed using TruSeq adapters followed by sequencing.

The current Illumina PE library sequencing protocol restricts library fragment size to 250–500 bp because longer fragments decrease the quality of data through overlapping and reduced density of clusters. Coverage of larger distances between nucleotide positions of interest can, however, be achieved through the mate paired (MP) library protocol. This protocol initially utilizes larger genomic fragments of 2–5 kb that are self-ligated prior to a secondary fragmentation to the conventional PE library size centering on 500 bp (Figure 1). Biotinylation of the termini of larger fragments prior to circularization enables the isolation of DNA containing the ligated ends. Sequencing of these fragments containing junction points thus generates paired reads that are linked across much greater distances than in conventional PE libraries, at the expense of some loss in coverage for short inter-variant distances. A combination of PE (100–600 bp) and MP (500–5,000 bp) libraries over a defined gene region could therefore complement each other in terms of phased coverage and should allow accurate determination of *cis*/*trans* status of multiple sequence variants over a relatively large range of distances.

We tested this supposition using the *CYP21A2* gene as a model system. This gene is commonly sequenced during diagnosis of congenital adrenal hyperplasia (CAH). The combination of the modest length of this gene (~3400 bp), a rate of at least 10% compound heterozygosity for mutations or variants of unknown significance in patients, and availability of genetic family studies in most cases, make *CYP21A2* a suitable model system as a proof of principle test of our approach.

## Methods

### Long-range PCR

**Figure 2:**
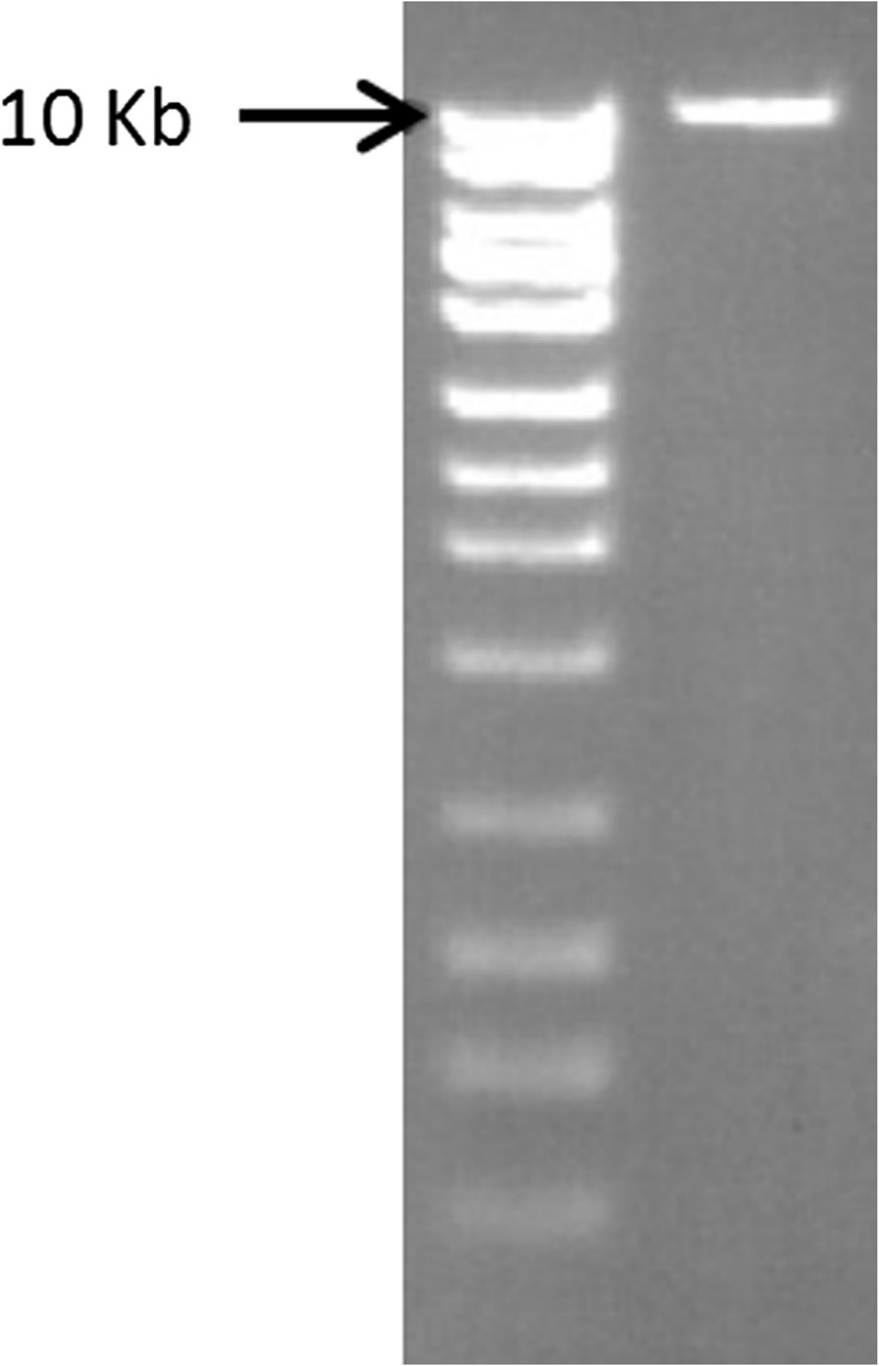
**Clean 10 Kb amplicons.** A single, clean band at 10 Kb shows the specificity of our long-range amplification.

*CYP21A2* is located in the HLA region on chromosome 6p2.13. An inactive yet highly homologous pseudogene (*CYP21A1P*) is located 30 kb upstream and has been known to confuse genotyping assays for *CYP21A2*[8,9]. To enrich our mate pair library with the active gene and eliminate the pseudogene we performed long-range PCR (lrPCR) using unique priming locations around *CYP21A2*. Priming sequences were 5’-AGTGGGGCTCTGAAGACTGA-3’ for the forward position and 5’-CCCTCGGGAGATGATCTGTA-3’ for the reverse to amplify a clean 10 kb product (Figure 2). LA Taq and associated buffers from TaKaRa were used in the reaction at their recommended concentrations. Approximately 150 ng of template DNA was used in the PCR reaction. Cycle conditions were as follows: 95°C for 5 m; 10 cycles of (95°C for 30 s, 60°C for 30 s, 72°C for 10 m); 20 cycles of (95°C for 30 s, 55 for 30 s, 72°C for 10 m); 72°C for 20 m.

### Whole genome amplified background DNA

The MP protocol is driven towards intra-molecular circularization over inter-molecular ligation of two separate DNA fragments simply by spatial dilution. In order to minimize the complication of inter-fragment ligations from a limited sequence amplicon input we investigated spiking of the 10 kb amplicon at four different concentrations into a background of whole genome amplified (WGA) DNA from a normal individual. WGA DNA was used due to its similar average fragment size to the 10 kb lrPCR amplicon, than conventional extracted genomic DNA preparations (~50 kb), making downstream fragmentation in the library prep protocol more predictable. This approach additionally enabled us to evaluate the role of amplicon concentration on inter-fragment ligation. Background WGA DNA was generated from genomic DNA using a Qiagen Repli-g midi kit according to recommended protocols. Background and amplified DNA concentrations were measured by fluorescence on a Qubit fluorometer (Invitrogen), and 10, 100, 500, and 1,000 ng aliquots of lrPCR product were spiked into WGA DNA to a total of 5 ug for each library preparation.

### Library preparation and sequencing

MP libraries were prepared for each spiked pool of lrPCR product and WGA background DNA based on previously reported protocols [10]. Each pool was fragmented on an E210 Focused-ultrasonicator (Covaris) to fragments ranging from 500 to 5000 bp with an average of 2000 bp. Following purification on Qiaex II beads, DNA fragment ends were repaired and biotinylated using a mixture of natural and biotinylated dNTPs. Excess reagents and by-products were removed using Qiaex II beads. Six-hundred ng of DNA from each pool were circularized in 16 hour ligation reactions at 30°C prior to exonuclease treatment at 37°C for 20 minutes to digest any remaining linear strands of DNA. The circularized DNA was then fragmented to 300–500 bp using the M220 Focused-ultrasonicator. Streptavidin beads were applied to isolate ligation junction fragments. End repair, blunt ending and adapter ligation were performed while fragments were bound to the beads. PCR was performed to produce bead-free fragments which were subsequently assembled into indexed MP libraries using TruSeq adapters (Illumina). While streptavidin beads provide good recovery of biotinylated DNA, they also co-purify a fraction of unlabeled fragments from other locations in the sheared, circularized DNA. We used this to our advantage by allowing these fragments into our libraries to provide PE reads covering positions 100 to 500 bp apart.

The four final indexed MP libraries were purified and analyzed on an Agilent Bioanalyzer DNA 1000 chip before equimolar pooling. The sample was loaded onto a single lane of an Illumina flow cell and sequenced to 101×2 paired-end reads on an Illumina HiSeq. Base calling was performed using Illumina Pipeline v1.5.

Sequence reads collected from the Illumina were demultiplexed and mapped to the hg19 assembly [11] using a custom mapping algorithm similar to the one used in previous publications [12,13]. To avoid the problem of reads from the amplified region erroneously mapping to the pseudogene, *CYP21A1P*, and/or homologous surrounding areas, the region from chromosome 6 between 31971000 and 31982000 was removed from the reference sequence.

### Statistical analysis

After mapping and alignment of linked reads covering two heterozygous positions a matrix was constructed to quantify the associations between every possible pair of base calls between the two positions. Confidence intervals for all base calls were calculated by bootstrapping based on the observed frequency of base calls in each association matrix. For each upstream base call (association matrix rows), a probability distribution was constructed for all possible downstream base calls (association matrix columns). Observed counts in each row were converted to probabilities and used for multinomial resampling with the total number of samples set to the sum of observations in the row. In addition to the observed probabilities, 1% was distributed across each row to simulate random error associated with NGS sequencing. Following every cycle of sampling, the counts for each base call were converted to probabilities and used to construct a set of distributions. After 1000 sampling iterations, confidence intervals were set for each possible downstream base call by ranking the resulting probabilities for that base and selecting the 1% and 99% values from the distribution.

For haplotyping regions longer than the span of PE or MP fragments several association matrices can be chained together. In this case, bootstrapping for each individual matrix was performed as described above. The linkage between pairs of heterozygous positions followed a Markov Chain model in that the probability of association between two base calls was unrelated to previous base calls in the chain. To begin the chain, association matrix *A*_*1*_ was constructed between two heterozygous positions, *h*_*0*_ and *h*_*1*_. One of the two bases was arbitrarily chosen from *h*_*0*_, and probabilities and confidence intervals for each base at *h*_*1*_ were calculated as described above. Next, association matrix *A*_*2*_ was constructed between positions *h*_*1*_ and *h*_*2*_. The base with highest probability at *h*_*1*_ from *A*_*1*_ was selected and probabilities for association with this base at *h*_*2*_ in *A*_*2*_ were calculated. By iteration of this cycle, a chain of associated base calls can easily be made for one allele. To validate the results from one allele, the opposite allele can be phased by selecting the alternate base at *h*_*0*_ and crosschecking the two resulting chains.

To quantify the confidence of association between two distant heterozygote calls, a cumulative probability was calculated as the product of all prior probabilities in the associated chain. Cumulative confidence intervals were also calculated from a distribution made from the products of each previously occurring bootstrap result. Using these measures, the limits to the length of chained phasing become apparent when the confidence intervals of rejected base calls begin to overlap with the cumulative interval.

## Results

To demonstrate that a combined PE and MP sequencing strategy could allow us to accurately phase compound heterozygous sequence variants over a significant genomic distance, we divided the problem into three components. First, we performed experiments to determine the necessary conditions for adequate sequence coverage and showed proof of principle of accurate variant phasing, using *CYP21A2* as a model system. Next, we demonstrated that the analysis can be extended to phase DNA fragments across distances that are much larger than those included in the MP library. Finally, we explored the principle sources of experimental error.

### Phasing a single pair of heterozygote sequence variants

Confidence of NGS base calls is a function of coverage at a given position. Since our strategy requires accurate association of two heterozygous positions (a total of 4 base calls), high coverage is required throughout the target region. To this end, we designed a long range-PCR (lrPCR) for enrichment by amplification of the active gene *CYP21A2*, while excluding its highly homologous pseudogene, *CYP21A1P*.

While enrichment boosts coverage, it also increases the likelihood that two fragments of DNA from opposite *CYP21A2* alleles will be ligated together during MP library construction. This event would generate false *cis* associations between loci. We reasoned that we could reduce the probability of inter-allelic recombination by adding an excess of background genomic DNA to the gene specific lrPCR product, biasing any recombination towards non-target sequences. Libraries made with 10, 100, 500 and 1000 ng of lrPCR product produced sequence coverages of 1,600×, 10,900×, 60,400× and 130,500×, respectively. By contrast, coverage by MP fragments outside of the amplified target region averaged slightly less than 2×.

**Figure 3:**
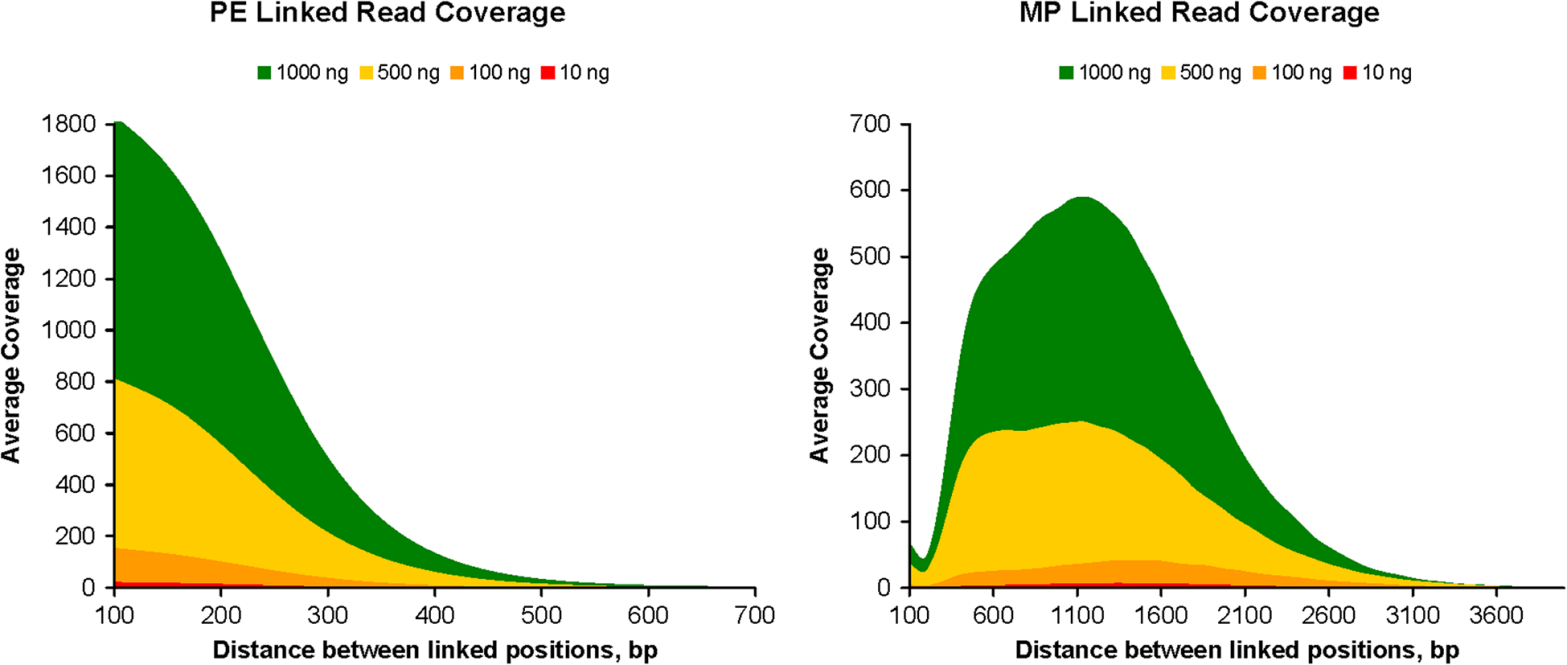
**Average linked coverage by PE and MP reads from four libraries.** Linked read coverage is shown from PE and MP reads as a function of distance between linked positions for each of the enriched libraries (10, 100, 500 and 1,000 ng spiked lrPCR amplicon in WGA DNA).

Linked reads are of even greater importance for phasing than raw coverage is for accuracy. Any linked read method for phasing needs to generate an extended distribution of fragment sizes. This assures enough depth of coverage between any two points within a gene to accommodate a broad range of potential distances between heterozygous positions. To verify that we had achieved this goal we calculated the linked coverage in our NGS data as a function of distance Δ between base positions. For every position *x* in the amplicon, we counted the number of linked reads covering both *x* and *x* + Δ, for Δ*s* from 101 to 3000 bp, and then calculated and plotted the average linked coverage. Paired end libraries provided linked reads up to 500 bp while MP libraries produced a population of fragments ranging from about 200 bp to over 3000 bp (Figure 3).

Previous genotyping of the specimen tested here showed two heterozygous disease-causing mutations; however their phase was not clear from the Sanger sequences and required family studies. The first mutation, c.60G > A introduces a stop codon at amino acid position 20. The second, IVS2-13A > G is a common splice site mutation in intron 2. Both variants produce truncated proteins and are associated with the classical form of CAH.

**Figure 4:**
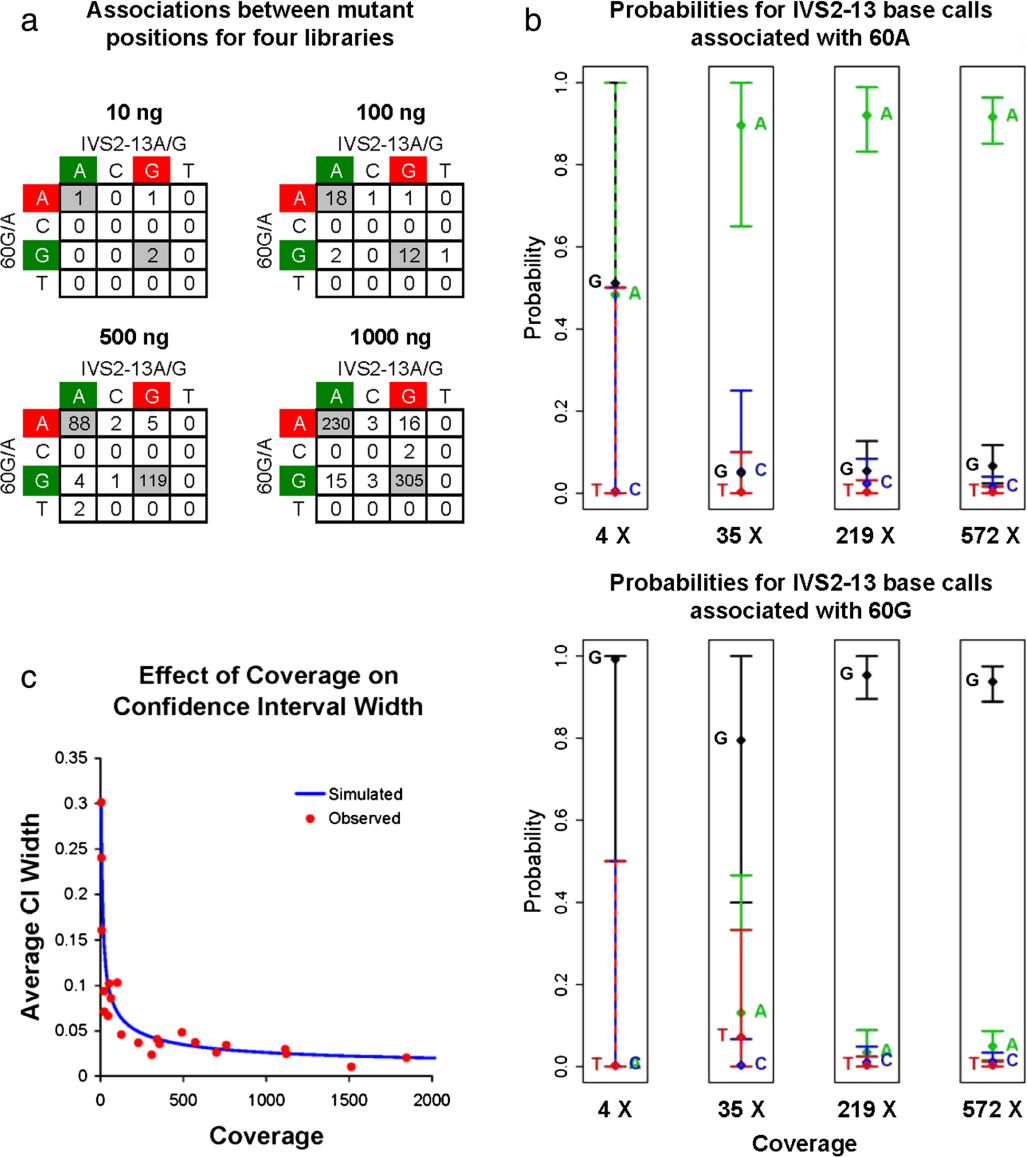
**Confidence in phasing calls is dependant on coverage. (a)** Association matrices from each spiked library show the relationship between the two disease-causing heterozygous positions in this specimen. Highlighting of the bases at each locus indicates wild-type (green) and mutant (red). **(b)** The probabilities and 99% confidence intervals for all possible IVS2-13 base calls associated with each c.60G/A allele are shown for all four amplicon spiked libraries. Coverage increases linearly as spike concentration increases. **(c)** Average confidence interval (CI) width was calculated to evaluate the level of coverage required for confident heterozygote association. A simulation run to test varying coverage and observed data points both indicate that coverage beyond 500× provides diminishing returns in CI width.

After mapping all of the reads in the library, fragments were selected that covered both heterozygous positions with their pairs of sequence reads. The base calls at each heterozygous position from each fragment were observed and compared to establish the relationship between the two alleles. Using these base calls, an association matrix was constructed to measure the frequency of each association (Figure 4a). In each library, the wild-type G at position c.60 was most frequently associated with a mutant G in the IVS2-13 position. Conversely, the mutant A at position c.60 was most frequently associated with the wild-type A at IVS2-13. This indicated that the two mutants were on opposite alleles, a result that was congruent with the conventional phased genotype that had previously been established through allelic segregation studies of the proband’s family.

The next step was to quantify more precisely how confident one could be that the *trans* phasing result was correct. We used bootstrapping for this, calculating 99% confidence intervals around the probability of each possible downstream base call. The width of the confidence intervals therefore, is related to the depth of linked coverage between the two mutant sites (Figure 4b). To clarify this relationship, we ran simulations for varying amounts of coverage using probabilities from a single dataset (500 ng amplicon spike) and calculated the average width of all resulting confidence intervals. Both the simulation and observed confidence intervals indicate that coverage above 500× provides diminishing returns in phasing confidence (Figure 4c).

### Extending the method over longer genetic distances

**Figure 5:**
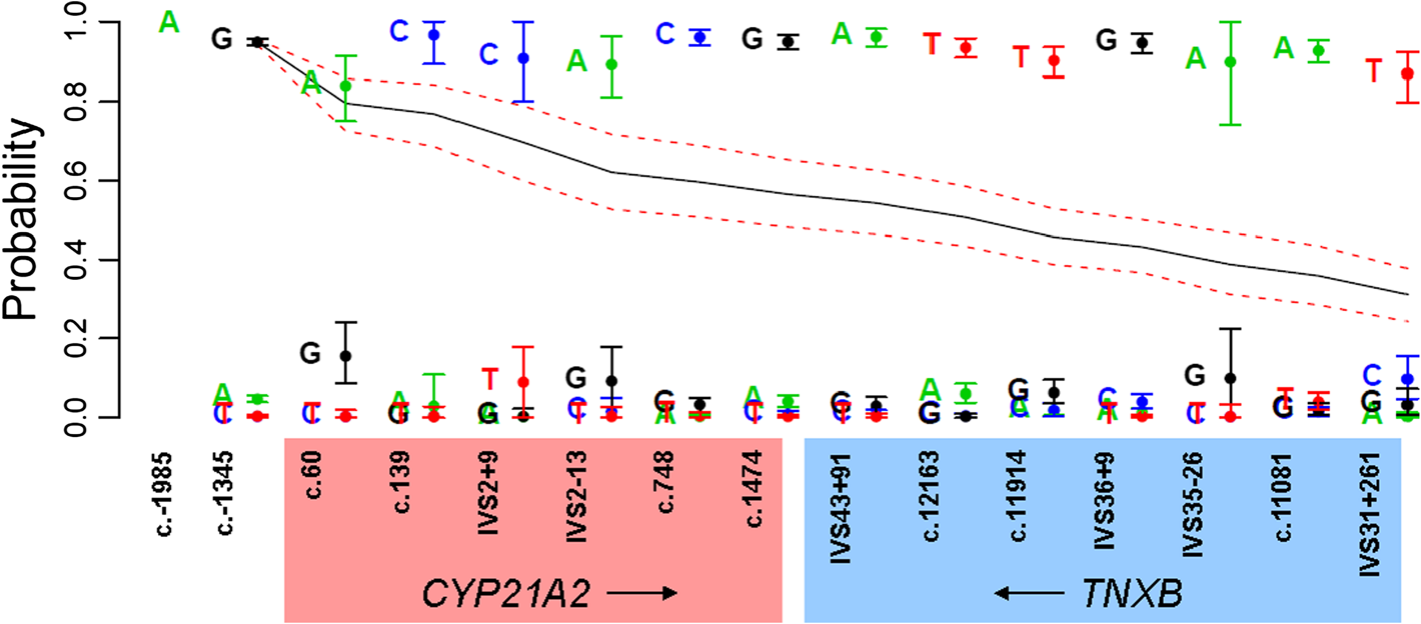
**One phase of the entire 10 kb amplicon.** By beginning with the first heterozygote in the amplicon and sequentially moving through all downstream heterozygous positions, the phase of the entire 10 kb amplicon can be determined. Confidence intervals in the columns show the relationship of each base to the highest probability base call from the previous column. Lines showing the cumulative probability and confidence interval relate each downstream position to the very first in the chain. Cumulative probability diminishes in proportion to the quality of each association matrix in the chain (i.e. sufficient coverage and few errors). However, one can be sure of the accuracy of phasing so long as there is no overlap between the cumulative confidence interval and that of any rejected base.

In our test specimen, the two mutant positions were well covered by a subset of PE and MP fragments. However, it is likely that in some cases (or in different genes) heterozygous mutations will be separated by more than 2000 bases. For these situations, we have developed a computational method to chain together linked reads by constructing association matrices between pairs of several heterozygous sequence positions (normal sequence variants, VUSs. or mutations) in tandem through the length of the amplified region. Provided there are enough heterozygous positions in the specimen that fall within the limits of the combined MP and PE libraries, the entire amplified region can be phased using this iterative approach. Statistical analysis of the phase assignment across an entire chain of linked sequence variants is identical to the single association matrix, except that a cumulative probability and confidence interval is calculated between the two mutant positions to measure confidence in the data used to link the two. Since this cumulative measure is the product of all upstream probabilities in the chain, its value will decline in proportion to the amount of error in each association matrix. This diminishes the probability of the final overall phase-call in relation to the first. However, as long as there is full separation of the confidence limits of the final cumulative phase determination from all other possibilities, a confident call can be made. It is thus possible to extend the phasing chain across the entire 10 kb lrPCR amplicon without any overlap of confidence intervals, indicating accurate phasing throughout (Figure 5).

### Sources of error in the association matrix

Each association matrix contains a small percentage of incorrectly linked bases. The sources of error in NGS datasets have been previously explored and attributed principally to detection error during data acquisition, fluorescence spectral overlap and computational misalignment of reads in highly homologous regions [14,15]. Since our protocol includes lrPCR enrichment followed by MP library preparation, we also had to consider the contribution from *in vitro* recombination events that occur during amplification or circularization.

While a thorough investigation of this type of error is beyond the scope of this paper, we were able to quantify two types of inaccuracy by constructing association matrices between every pair of heterozygotes in the *CYP21A2* gene. Recombination events, i.e. MP reads that include a base from either allele, were the most common source of error. As a percentage of total reads, this type of error averaged about 7% and it proved to be constant across every combination of PCR product and background DNA mix (5.4%, 7.4%, 6.2% and 7.2% error for 10, 100, 500 and 1000 ng of amplicon input, respectively). In addition, there was no change in these error rates as a function of linked read length or coverage. This indicates that our initial assumption was incorrect; ligation of fragments from opposite alleles during MP library preparation did not prove to be a major contributor to erroneous base or phasing calls. Furthermore, because the percentage of recombination does not change with proportion to the amount of amplicon spiked into each library, these events must occur prior to library creation, i.e. during lrPCR. These observations testify to the reliability of the MP library protocol and highlight the importance of high fidelity in PCR reactions.

Finally, some incorrect base and/or phasing call errors could not be attributed to recombination artefacts. Across all heterozygous pairs analyzed in our data, only 2% of the total reads fell into this category, a value that accords with other reported values for random error in NGS data [16,17].

## Discussion

Using our MP library approach and subsequent computational analysis we have been able to successfully haplotype a specific region of interest in an individual who had two heterozygous disease-causing mutations. This method is an improvement over other available phasing protocols because of its simplicity and because of the statistical measure of assurance it provides. In regions where coverage is low or where recombination is present in the fragment library, erroneous phasing calls can easily be made by other methods. In addition to these advantages, our method provides the ability to phase heterozygous positions that are thousands of bases apart.

Performed as a single protocol, this method is capable of acquiring a completely phased genotype for an entire 10 kb lrPCR amplicon. Target regions of this size can be routinely amplified, and 20–30 kb amplicons are achievable in many instances. In principle, this method could also work beyond 10–30 kb, if several overlapping lrPCR amplicons are used as starting material, and as long as sufficient overlapping MP fragments can be generated that share heterozygous positions. An average MP library size which exceeds the 2 kb observed in our study would be expected to improve the likelihood of finding an unbroken linked chain of polymorphisms, while simultaneously reducing the number association matrices needed for complete phasing of a region of interest, thereby improving the confidence in the accuracy of the overall haplotype. Since the Illumina MP protocol is optimized for initial fragmentation libraries of 2–5 kb, such improvements should be relatively easy to achieve.

In theory, there is no upper limit to the scalability of our approach and it could even be applied to whole genome sequencing, provided sequence coverage and linked coverage are high enough. Without regard to logistic or cost considerations, we speculate that this technique might actually be very successful in this setting, because the error attributable to inter-allelic MP ligation proved to be very low. Nevertheless, it is likely that one would have to break down the analysis of an entire genome into smaller haplotype units, in order to maintain high confidence of the phase calls. We would anticipate that the size of these units would be similar to what can be achieved by optimal combinations of lrPCR and MP protocols, as described above.

Two limitations that we foresee for accurate phasing are highly homologous genes and gene duplications or other copy number changes. In either of these cases, we would anticipate phasing errors to increase due to mis-assignment of reads. In addition, increases in gene copy number would exponentially increase the number of possible phase combinations for any given combination of polymorphic positions, increasing computational requirements and decreasing ultimate haplotyping accuracy, and in some cases, phase assignments might be impossible.

## Conclusions

In summary, compared with previous approaches, our MP NGS sequencing technique is a simple solution to the problem of accurately phased genotyping for many recessive diseases, and perhaps, many other genetic phasing problems. The method could be adapted to other NGS platforms since they are all based on deriving sequences by aligning large numbers of overlapping reads. As clinical molecular diagnosis rapidly approaches massively parallel sequencing as the preferred assay method, it could serve as a cost-effective way to obtain a completely resolved set of haplotypes for single genes, panels of related genes, or even significant portions of chromosomes.

## Authors’ contributions

KWC, SJM, and RAS performed molecular biology and sequencing experiments. TMD and GV provided mapping and informatics. CN, NLE, SKGG, GV, and KWC provided statistical analysis and data interpretation. KWC wrote the algorithms and drafted the manuscript. All authors read and approved the final manuscript.

## Pre-publication history

The pre-publication history for this paper can be accessed here:

http://www.biomedcentral.com/1471-2350/15/19/prepub

## References

[B1] Online Mendelian Inheritance in Man, OMIM® World Wide Web[http://omim.org/]

[B2] Salem R Wessel J Schork N A comprehensive literature review of haplotyping software and methods for use with unrelated individuals Hum Genomics 2005 15 39 66 10.1186/1479-7364-2-1-39 15814067 PMC3525117

[B3] Yan H Papadopoulos N Marra G Perrera C Jiricny J Boland CR Lynch HT Chadwick RB de la Chapelle A Berg K Conversion of diploidy to haploidy - individuals susceptible to multigene disorders may now be spotted more easily Nature 2000 15 723 724 10.1038/35001659 10693791

[B4] Douglas JA Boehnke M Gillanders E Trent JM Gruber SB Experimentally-derived haplotypes substantially increase the efficiency of linkage disequilibrium studies Nat Genet 2001 15 361 364 10.1038/ng582 11443299

[B5] Fan HC Wang J Potanina A Quake SR Whole-genome molecular haplotyping of single cells Nat Biotechnol 2011 15 51 57 10.1038/nbt.1739 21170043 PMC4098715

[B6] Kitzman JO Mackenzie AP Adey A Hiatt JB Patwardhan RP Sudmant PH Ng SB Alkan C Qiu R Eichler EE Shendure J Haplotype-resolved genome sequencing of a Gujarati Indian individual Nat Biotechnol 2011 15 59 63 10.1038/nbt.1740 21170042 PMC3116788

[B7] Kaper F Swamy S Klotzle B Munchel S Cottrell J Bibikova M Chuang H-Y Kruglyak S Ronaghi M Eberle MA Fan J-B Whole-genome haplotyping by dilution, amplification, and sequencing Proc Natl Acad Sci 2013 15 5552-5557 5557 23509297 10.1073/pnas.1218696110 PMC3619281

[B8] Tsai LP Cheng CF Chuang SH Lee HH Analysis of the CYP21A1P pseudogene: indication of mutational diversity and CYP21A2-like and duplicated CYP21A2 genes Anal Biochem 2011 15 133 141 10.1016/j.ab.2011.02.016 21324303

[B9] Concolino P Mello E Zuppi C Capoluongo E Molecular diagnosis of congenital adrenal hyperplasia due to 21-hydroxylase deficiency: an update of new CYP21A2 mutations Clin Chem Lab Med 2010 15 1057 1062 20482300 10.1515/CCLM.2010.239

[B10] Murphy SJ Cheville JC Zarei S Johnson SH Sikkink RA Kosari F Feldman AL Eckloff BW Karnes RJ Vasmatzis G Mate pair sequencing of whole-genome-amplified DNA following laser capture microdissection of prostate cancer DNA Res 2012 15 395 406 10.1093/dnares/dss021 22991452 PMC3473372

[B11] Lander ES Consortium IHGS Linton LM Birren B Nusbaum C Zody MC Baldwin J Devon K Dewar K Doyle M Initial sequencing and analysis of the human genome Nature 2001 15 860 921 10.1038/35057062 11237011

[B12] Vasmatzis G Johnson SH Knudson RA Ketterling RP Braggio E Fonseca R Viswanatha DS Law ME Kip NS Ozsan N Genome-wide analysis reveals recurrent structural abnormalities of TP63 and other p53-related genes in peripheral T-cell lymphomas Blood 2012 15 2280 2289 10.1182/blood-2012-03-419937 22855598 PMC5070713

[B13] Feldman AL Dogan A Smith DI Law ME Ansell SM Johnson SH Porcher JC Ozsan N Wieben ED Eckloff BW Vasmatzis G Massively parallel mate pair DNA library sequencing for translocation discovery: recurrent t(6;7)(p25.3;q32.3) Translocations in ALK-negative anaplastic large cell lymphomas Blood 2010 15 278 278 10.1182/blood-2010-08-303305 PMC3035081 21030553

[B14] Kircher M Stenzel U Kelso J Improved base calling for the illumina genome analyzer using machine learning strategies Genome Biol 2009 15 10.1186/gb-2009-10-8-r83 PMC2745764 19682367

[B15] Nakamura K Oshima T Morimoto T Ikeda S Yoshikawa H Shiwa Y Ishikawa S Linak MC Hirai A Takahashi H Sequence-specific error profile of Illumina sequencers Nucleic acids research 2011 15 e90 10.1093/nar/gkr344 21576222 PMC3141275

[B16] Luo CW Tsementzi D Kyrpides N Read T Konstantinidis KT Direct comparisons of illumina vs. Roche 454 sequencing technologies on the same microbial community DNA sample Plos One 2012 15 10.1371/journal.pone.0030087 PMC3277595 22347999

[B17] Glenn TC Field guide to next-generation DNA sequencers Mol Ecol Resour 2011 15 759 769 10.1111/j.1755-0998.2011.03024.x 21592312

